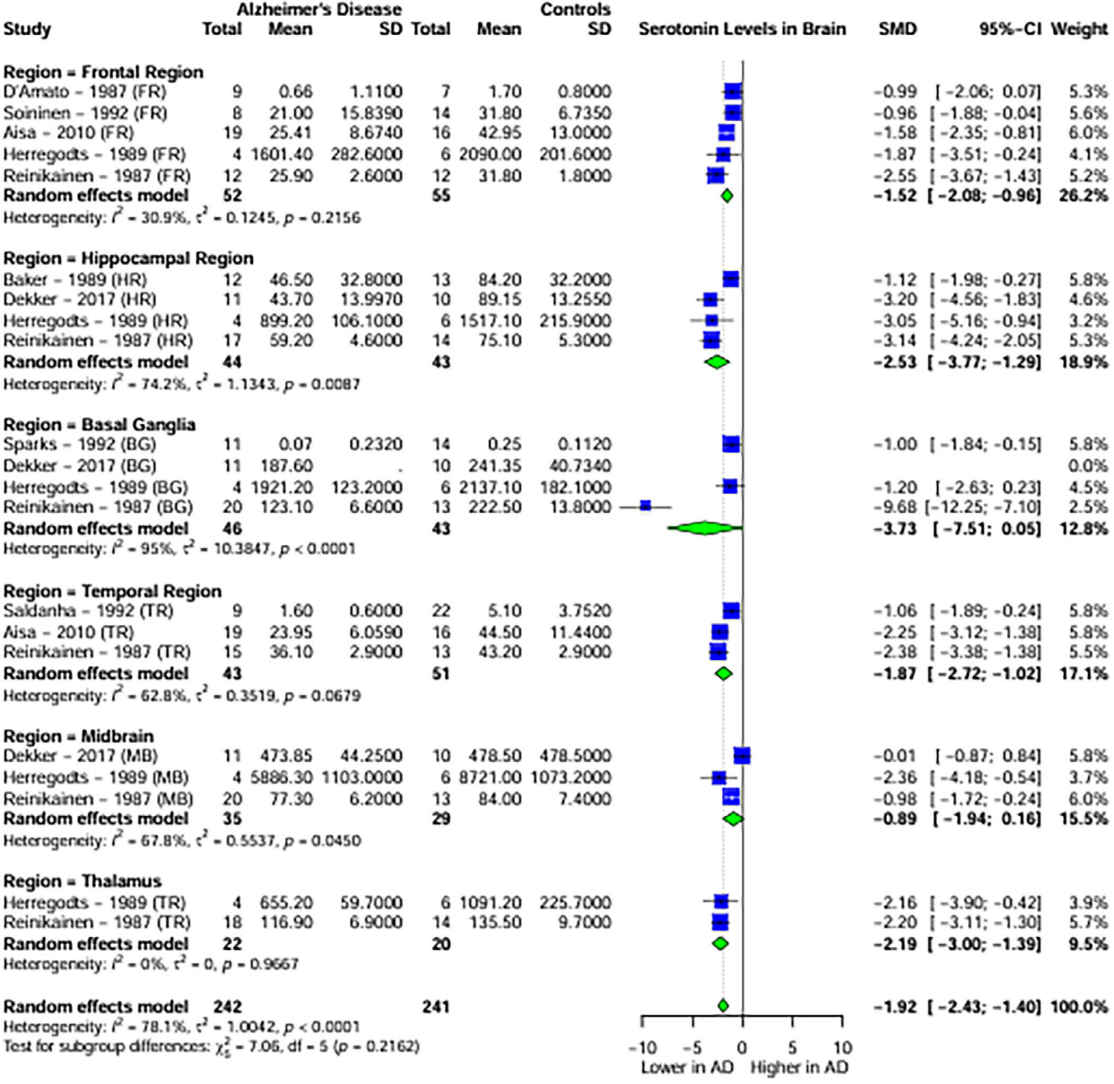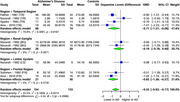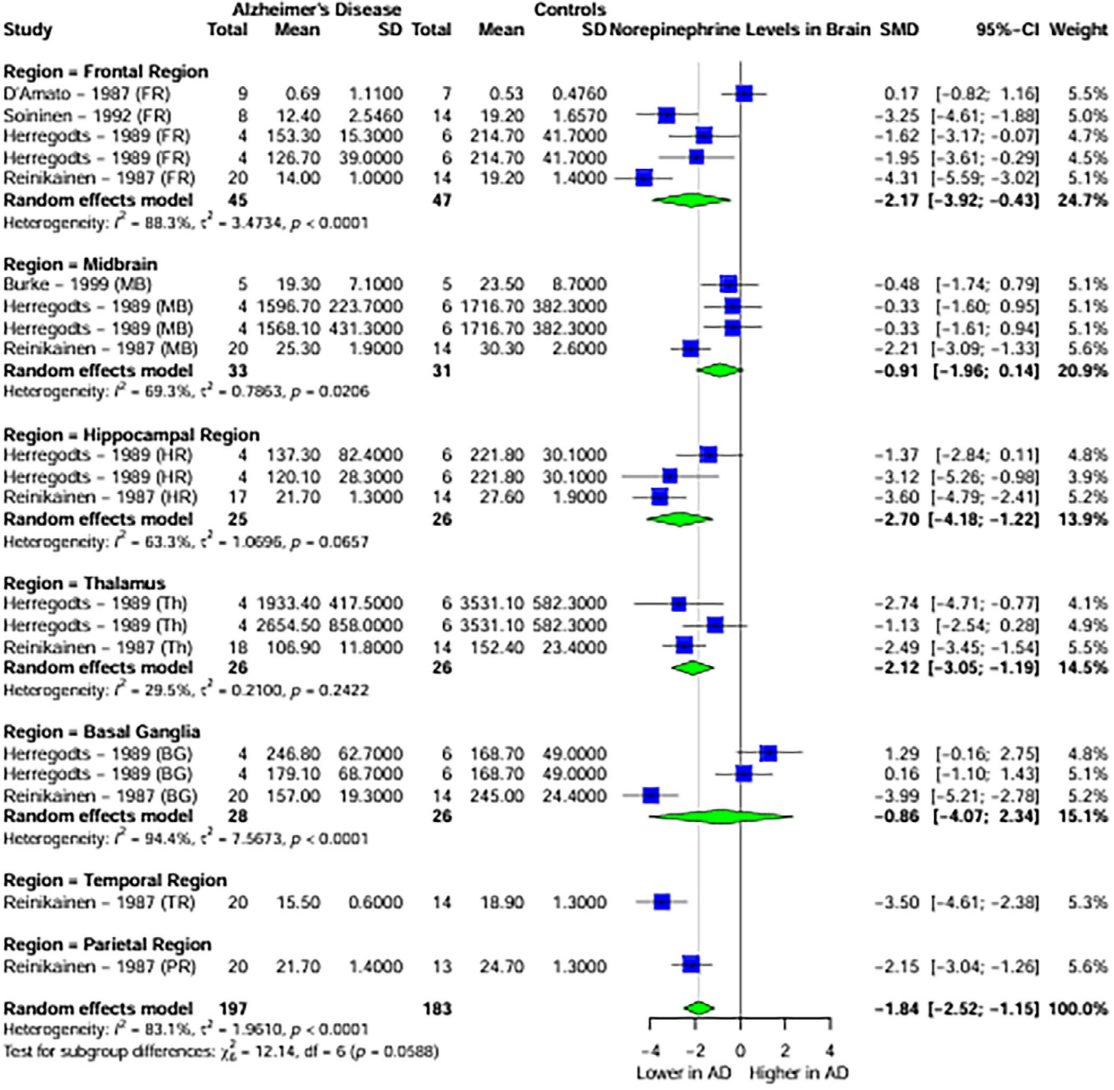# Neurotransmitter Dysregulation in Alzheimer's Disease: A Meta‐Analysis of Serotonin, Dopamine, and Norepinephrine Alterations Across Brain Regions

**DOI:** 10.1002/alz70862_110161

**Published:** 2025-12-23

**Authors:** Muneeb Ahmad Muneer, Harshita Agarwal, Poorvikha Gowda, Mohammad Orooj Azmi, Parshant Yadav, Victor Ghosh, Mohammad Hassan, Anmol Kaur, Vinay Suresh, Ahmed Y. Azzam, Mainak Bardhan

**Affiliations:** ^1^ Allama Iqbal Medical College, Lahore, Punjab Pakistan; ^2^ Institute of Post Graduate Medical Education and Research, Kolkata, West Bengal India; ^3^ St John’s Medical College, Bangalore, Karnataka India; ^4^ Maulana Azad Medical College, New Delhi, Delhi India; ^5^ Andhra Medical College, Visakhapatnam, Andhra Pradesh India; ^6^ Katihar Medical College, Katihar, Bihar India; ^7^ Lady Hardinge Medical College, New Delhi, Delhi India; ^8^ Research Peer Network ‐ Neurology Study Group, Lucknow, Uttar Pradesh India; ^9^ Montefiore‐Einstein Cerebrovascular Research Lab, Albert Einstein College of Medicine, New York, NY USA; ^10^ Miami Cancer Institute, Baptist Health South Florida, USA, Miami, FL USA

## Abstract

**Background:**

Alzheimer's disease (AD) is a complex disorder involving neuroinflammation, vascular impairment, and synaptic dysfunction. This meta‐analysis aims to evaluate and compare dopamine, serotonin, and norepinephrine levels across brain regions in AD patients and healthy controls, exploring their potential as early diagnostic biomarkers.

**Method:**

We searched MEDLINE, EMBASE, Cochrane, and Scopus for studies on dopamine, serotonin, and norepinephrine concentrations in AD patients, following PRISMA guidelines. The meta‐analysis utilized R’s 'meta' package to calculate standardized mean differences (SMD) and assess heterogeneity using I² and tau².

**Result:**

Our comprehensive analysis of multiple brain tissues revealed significant alterations in specific brain regions of AD patients compared to healthy controls. Serotonin exhibited a substantial SMD of ‐1.92 (95% CI: ‐2.43 to ‐1.40, I² = 78.1%) across 9 studies (242 AD, 241 controls), with the greatest reduction in the hippocampal region (SMD: ‐2.53, 95% CI: ‐3.77 to ‐1.29), followed by the thalamus (SMD: ‐2.19, 95% CI: ‐3.00 to ‐1.39), temporal region (SMD: ‐1.87, 95% CI: ‐2.72 to ‐1.02), and frontal region (SMD: ‐1.52, 95% CI: ‐2.08 to ‐0.96). Dopamine showed a significant overall SMD of ‐0.55 (95% CI: ‐0.93 to ‐0.17, I² = 55.5%) across 5 studies (154 AD, 133 controls), with the most pronounced reductions in the frontal (SMD: ‐0.82, 95% CI: ‐1.38 to ‐0.27) and temporal regions (SMD: ‐0.71, 95% CI: ‐1.37 to ‐0.06). Norepinephrine exhibited a notable SMD of ‐1.84 (95% CI: ‐2.52 to ‐1.15, I² = 83.1%) in 5 studies (197 AD, 183 controls), with the greatest reductions in the hippocampal region (SMD: ‐2.70, 95% CI: ‐4.18 to ‐1.22), frontal region (SMD: ‐2.17, 95% CI: ‐3.92 to ‐0.43), and thalamus (SMD: ‐2.12, 95% CI: ‐3.05 to ‐1.19).

**Conclusion:**

Serotonin, dopamine, and norepinephrine levels were significantly reduced in specific brain regions in AD. The most substantial decrease in serotonin was observed in the hippocampus, thalamus, temporal, and frontal regions. Dopamine levels were notably reduced in the temporal and frontal regions, while norepinephrine showed significant reductions in the hippocampus, thalamus, and frontal regions. Further validation of these findings is required.